# Blood pressure in atrial fibrillation and in sinus rhythm during ambulatory blood pressure monitoring: data from the TEMPLAR project

**DOI:** 10.1038/s41440-023-01473-x

**Published:** 2023-10-23

**Authors:** Kristina Lundwall, Thomas Kahan, Stefano Omboni

**Affiliations:** 1 Division of Cardiovascular Medicine, Department of Clinical Sciences, Danderyd Hospital, Karolinska Institutet, Stockholm, Sweden; 2 Clinical Research Unit, Italian Institute of Telemedicine, Varese, Italy; 3 Department of Cardiology, Sechenov First Moscow State Medical University, Moscow, Russian Federation

**Keywords:** Ambulatory blood pressure monitoring, Hypertension, Atrial fibrillation, Sinus rhythm, Intra individual comparisons

## Abstract

The coexistence of hypertension and atrial fibrillation (AF) is common and accounts for a worse prognosis. Uncertainties exist regarding blood pressure (BP) measurements in AF patients by automated oscillometric devices. The Microlife WatchBP 03 AFIB ambulatory BP monitoring (ABPM) device including an AF algorithm with each measurement was used in 430 subjects aged >65 years referred for ABPM and with assumed paroxysmal AF to perform intra-individual comparisons of BP during both AF-indicated and sinus rhythm. Only subjects with >30% of measurements indicating AF and episodes >30 min for assumed AF and for sinus rhythm were included. Mean age was 78 ± 7 years, 43% were male, 77% hypertensive, and 72% were treated. Compared to sinus rhythm, 24-h mean arterial pressure was similar (87.2 ± 9.5 vs 87.5 ± 10.6 mm Hg, *p* = 0.47), whereas 24-h systolic BP tended to be lower (123.6 ± 13.9 vs 124.7 ± 16.1 mm Hg, *p* = 0.05) and night-time diastolic BP higher (64.6 ± 10.9 vs 63.3 ± 10.4 mm Hg, *p* = 0.01) in assumed AF. Diastolic (not systolic) BP variability was higher in AF (*p* < 0.001). Results were similar with heart rates <90 and ≥90 bpm. In conclusion, this is the first study to use intra-individual comparisons of averaged BP during an ABPM in assumed paroxysmal AF and sinus rhythm. Our results imply that ABPM is feasible and informative also in patients with AF. We also suggest that an AF detection algorithm offers a new approach to evaluate the reliability of averaged BP values in AF compared to SR during an ABPM.

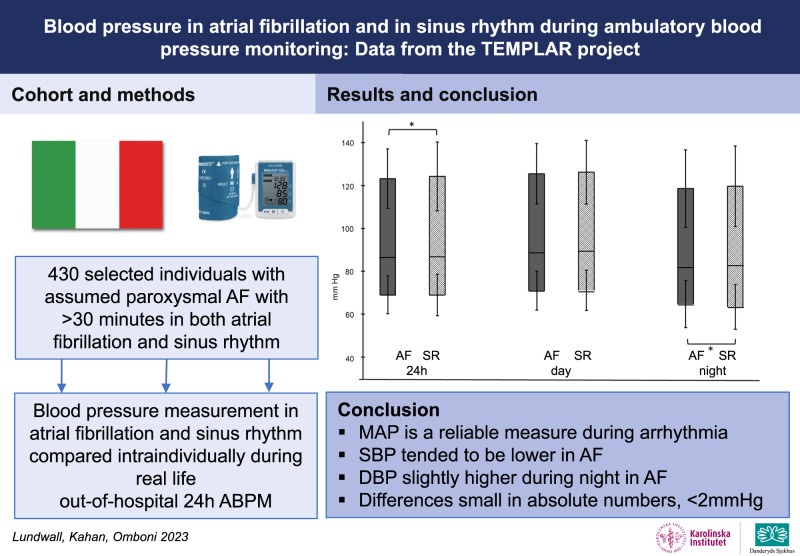

## Introduction

Atrial fibrillation (AF) is the most prevalent arrhythmia, and accounts for both increased morbidity and mortality. Hypertension, the most common risk factor for AF, is the leading cause of cardiovascular morbidity and mortality worldwide [1, 2]. Already a modest elevation in blood pressure (BP) increases the risk for incident AF [3, 4], and concomitant hypertension and AF accounts for a multiplied cardiovascular risk enhancement [5]. Lowering BP is the major factor for reducing risk for cardiovascular morbidity and mortality, and patients with the combination of AF and hypertension are therefore in need of optimal BP control.

As patients with AF generally have not been included in available cohort studies, it has been debated whether the prognostic information on CV events provided by BP measurements is similar in an AF population as in patients with SR; but this seems to be the case [6]. According to current practice guidelines, home BP and 24 h ambulatory BP monitoring (ABPM) are recommended methods for the diagnosis and treatment of hypertension [7]. Out-of-office methods of BP measurement may offer superior risk assessment also in the AF population, where studies have shown a stronger correlation between both left ventricular hypertrophy and left atrial size to ABPM than to office BP [8].

However, uncertainties regarding the accuracy of BP measurements in patients with ongoing AF have been emphasized, especially for automated devices, which are used for home BP and ABPM [9], and general practice guidelines for the use of automated devices in AF are lacking [7, 10]. Two meta-analyses comparing manual (mercury or aneroid) sphygmomanometers to automated (oscillometric or automated Korotkoff) home BP and ABPM devices in AF showed a reasonably good conformity between measurements in pooled analyses [8, 11]. However, all studies included were performed in a hospital setting, comparing short sets of measurements at rest. In addition, considerable heterogeneity according to type and brand of device, and small samples in the studies, did not allow for general recommendations and the authors emphasized the need for validation of out-of-office BP measurement in AF [8, 11]. Of note, inter-observer and intra-observer variability of BP measurements with mercury sphygmomanometers were reported greater during AF than in sinus rhythm (SR), leaving doubts about the manual sphygmomanometers as reference method in AF [12]. BP may change during AF, as compared to SR, due to hemodynamic alterations. Some studies comparing BP in AF and in SR in the same subjects before and after direct current cardioversion to restore SR suggest a trend for higher systolic and lower diastolic BP in SR [13–16]. However, results are inconsistent, study populations were small, and comparisons were not made to intra-arterial BP. One recent study comparing intra-aortic BP to brachial BP assessed by an oscillometric method showed a larger difference to invasive measurements in patients in AF than in those in SR [17]. This suggests differences in BP to be more dependent on technical than on hemodynamic issues.

We have found no studies on the accuracy of BP measurements in AF patients in an out-of-hospital setting, where most of the automated BP measurements are performed to guide therapy [8, 11]. However, the development of AF detection algorithms implemented in automated BP devices offer new opportunities. Several studies have investigated the accuracy of the Microlife Afib detection algorithm [8, 18–20], demonstrating a pooled sensitivity of 95% (95% confidence interval 92–98%) and a specificity of 94% (92–96%) for detecting true AF. False positives were almost exclusively frequent supraventricular extrasystolic beats [8].

Thus, in this study we performed out-of-hospital ABPM recordings with a device having the Microlife Afib detection algorithm incorporated. We investigated differences in averaged BP variables in the same subjects during episodes of both paroxysmal AF and SR during a full ABPM recording. Second, we aimed to evaluate if heart rate and low vs high BP during AF and SR could influence the results, and lastly if BP variability was dependent on rhythm.

## Methods

### Study design and population

This is a report from the TEMPLAR project, a multicenter, observational, cross-sectional study performed in community pharmacies in Italy. Details on the study design can be found elsewhere [21, 22]. In short, patients of either sex, referred by a general practitioner with a guideline based clinical indication for ABPM recording were eligible. In the current analysis, patients with ABPM investigations from November 2014 to December 2020 were included, provided the participants were aged ≥65 years, a measurement was made with a device incorporating the Microlife Afib detection algorithm, and the ABPM recording was valid according to established criteria (i.e., >70% successful readings, including 20 valid readings during the day and at least 7 valid readings during the night) [23]. Informed consent was obtained, and information about treatments and concomitant diseases were collected by the pharmacist during fitting or removal of the ABPM. Thus, previous diagnoses and concomitant diseases were patient reported or indicated by ongoing reported medications. The study was conducted in accordance with the declaration of Helsinki and the European General Data Protection Regulation (GDPR) and was registered in ClinicalTrials.gov (NCT03781401) and the Registry of Patient Registries (RoPR; No 41818). Ethical approval was waived since all examinations were performed as part of clinical praxis.

### Procedures for ambulatory blood pressure monitoring

Procedures for the ABPM recordings have been reported [21, 22]. In short, a validated oscillometric ABPM device with an algorithm incorporated to detect irregular rhythm and possible AF during BP measurement was used (Microlife WatchBP 03 AFIB, Microlife AG, Widnau, Switzerland) [18–20, 24–26]. ABPMs were fitted on the non-dominant arm by a trained pharmacist, and patients were asked to keep to daily routines and to complete a diary of activities and sleeping hours. BP measurement intervals were set to a standard of four times per hour (range of one to five times per h). During ABPM recording, BPs during irregular rhythm, falling into the AF algorithm criteria [27], were marked by the device as possible AF. At time of removal of the ABPM, the pharmacist uploaded ABPM data as well as patient characteristics to a dedicated telemedicine platform [28].

### Processing of data

All data was cleaned from artifacts and invalid recordings according to recommendations [29] before analyses. To account for confounding influence on BP variability indices from patients performing more than 24 h recording, a maximum number of 192 BP measurements for each participant was allowed, corresponding to a maximum of 48 h ABPM for each participant in the analysis.

#### Selection of measurements during assumed atrial fibrillation and sinus rhythm

First, individuals with >30% of measurements indicating AF were selected. This cut off was used previously in validation studies comparing the accuracy of the Microlife Afib algorithm to Holter ECG recordings to exclude subjects with frequent supraventricular extrasystolic beats [18, 19]. Second, at least three consecutive BP measurements in each rhythm was required, with the rational of discarding measurements during episodes shorter than 30 min for each rhythm, where 3 readings equals 36 min with the maximum of five BP readings per hour (see above for the setting of BP measurement intervals). This discarded individuals without longer episodes of both rhythms present during the recording, such as patients with permanent AF. It also further excluded measurements during supraventricular extrasystolic beats, and achieved episodes of sustained BP in each rhythm. Thus, individuals with >30% of measurements in AF-indicated rhythm during a period of 24–48 h, where episodes included were >30 min of duration, were assumed to represent measurements during AF. According to previous validation studies using a cut off >30% of measurements during one ABPM rendered false positive cases in 6–20% [8, 18, 19]. Remaining measurements without indicated AF were assumed to be in SR.

#### Blood pressure variables and heart rate

Individual systolic and diastolic BP, mean arterial pressure (MAP) and pulse rate (which was taken to represent heart rate, HR), during assumed AF and SR were collected. Daytime and night-time measurements were defined by recorded sleeping hours from the diary of daily activities. Pulse pressure was calculated as systolic BP – diastolic BP. Variability was assessed with averaged real variability (ARV) using the mean of absolute differences between adjacent BP values, as this was considered most appropriate for assessing short term BP variability [30, 31].The coefficient of variation (CV) of ARV (ARV/mean) was calculated to take mean values into account when assessing the dispersion of data. Data was then averaged for 24-h, daytime, and night-time mean values in AF and in SR.

#### Measurements according to heart rate <90 and ≥90 bpm

Analyses were also performed to account for the potential impact of HR on BP. We found few previous studies [17, 32], which evaluated the impact of HR on BP measurements in AF. Guided by these results and by the few cases in this study with a HR > 100 bpm (i.e., the prevailing clinical definition of tachycardia), the partition value for high HR was set to ≥90 bpm. Individual averaged mean values of systolic and diastolic BP, MAP, and PP during 24-h, daytime and night-time measurements with HR < 90 and ≥90 bpm were accordingly calculated for assumed AF and SR.

#### Measurements according to average blood pressure

A potential factor for individual rhythm dependent differences in BP is high vs low BP. To account for this, BP values were stratified by the average value (< or *≥*124 mmHg) for 24 h systolic BP, and average value (< or *≥*64 mmHg) for night-time diastolic BP, the two BP variables with trends for rhythm dependent differences. BPs were then compared intra-individually between AF and SR within these BP groups.

### Statistical analysis

Data are presented as mean values ± SD or median values with IQR, or numbers and frequencies (*n*; %), as appropriate. Power calculations for paired comparisons showed the need of 100 subjects (80% power, α 0.05). Rhythm dependent intraindividual differences in BP variables were investigated with Student’s paired *t*-tests. To account for the impact on variability indices by the number of repeated BPs, and the number of AF and SR measurements for each individual, a regression analysis was performed. BP in SR was used as dependent, BP in AF as independent, and number of BP measurements as well as the percentage of AF measurements as covariates. Three potential moderators were acknowledged: sex, age (65–75, 76–85, or *≥*86 years), and ongoing antihypertensive medication. Repeated measures ANOVA with interaction terms were performed to investigate the impact of these moderators. If interaction terms were significant, data was split by that moderator and analyzed with groups separated. Similar analyses were performed for BP with HR < 90 and ≥90 bpm. The significance level was set to a two-sided probability (*p*) value of <0.05. SPSS version 27 (IBM Corp. IBM SPSS Statistics for Windows. Armonk, NY, USA) was used for the analyses.

## Results

### General

**Fig. 1:**
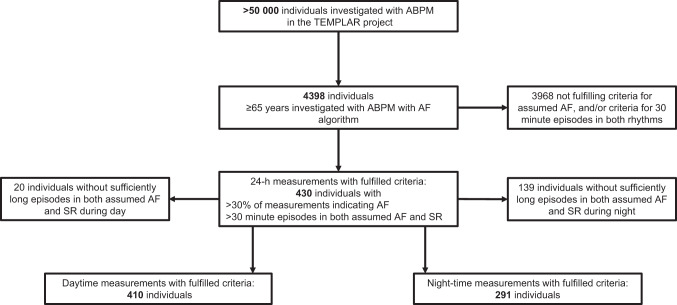
Inclusion and exclusion flow chart. ABPM ambulatory blood pressure monitoring, AF atrial fibrillation, SR sinus rhythm. The ABPM device with AF algorithm used was the Microlife WatchBP 03 AFIB ABPM, indicating rhythm with each blood pressure measurement

From 4398 eligible subjects the device indicated AF in >30% of the measurements during 24–48 h performed ABPMs for each subject in 456 individuals. Episodes >30 min in both SR and in assumed AF were present in 430 subjects, who were subsequently included in the analyses (Fig. 1). Mean age was 78 ± 7 years, 43% were male, 77% had a previous diagnosis of hypertension, and 72% were on antihypertensive treatment. Cardiovascular disease was present in 43%, hyperlipidemia in 26%, and diabetes mellitus in 9%; only 3.5% were previously diagnosed with AF.

Among the 430 participants, 37 performed ABPM for 48 h. Number of BP measurements per individual varied between 37 and 192 (mean 87 ± 23.2) for the full 24–48 h measurement, between 25 and 144 (62 ± 15.9) for daytime and between 8 and 84 (25 ± 10.7) for night-time measurements. A majority had four measurements per h, however two measurements per h during night-time was also common. Only a few subjects had episodes with one or five measurements per h. For each participant, the proportion of measurements in AF (after discarding AF indicated measurements <30 min) was calculated as the ratio of AF-indicated BP measurements to the total number of BP measurements performed for that individual. Median values were 32.0 [20.0–56.6], 31.1 [16.6–53.4], and 37.5 [21.4–62.5] % during 24-h, daytime, and night-time, respectively.

### Blood pressure values, according to rhythm

**Fig. 2:**
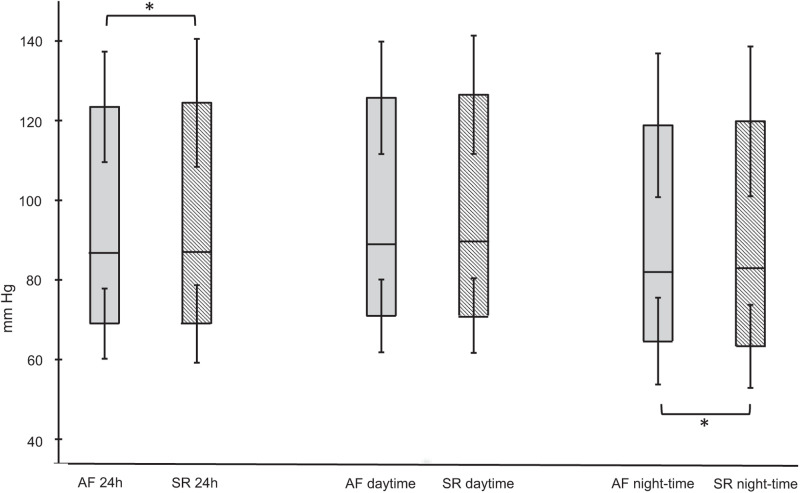
Blood pressure variables during 24-h, daytime, and night-time ABPM measurements. Upper and lower bar ends represent mean systolic and diastolic blood pressures ±SD, while vertical bars represent mean values of mean arterial pressure. AF atrial fibrillation, SR sinus rhythm. * denotes *p* < 0.05 for intraindividual differences between assumed AF and SR

**Fig. 3:**
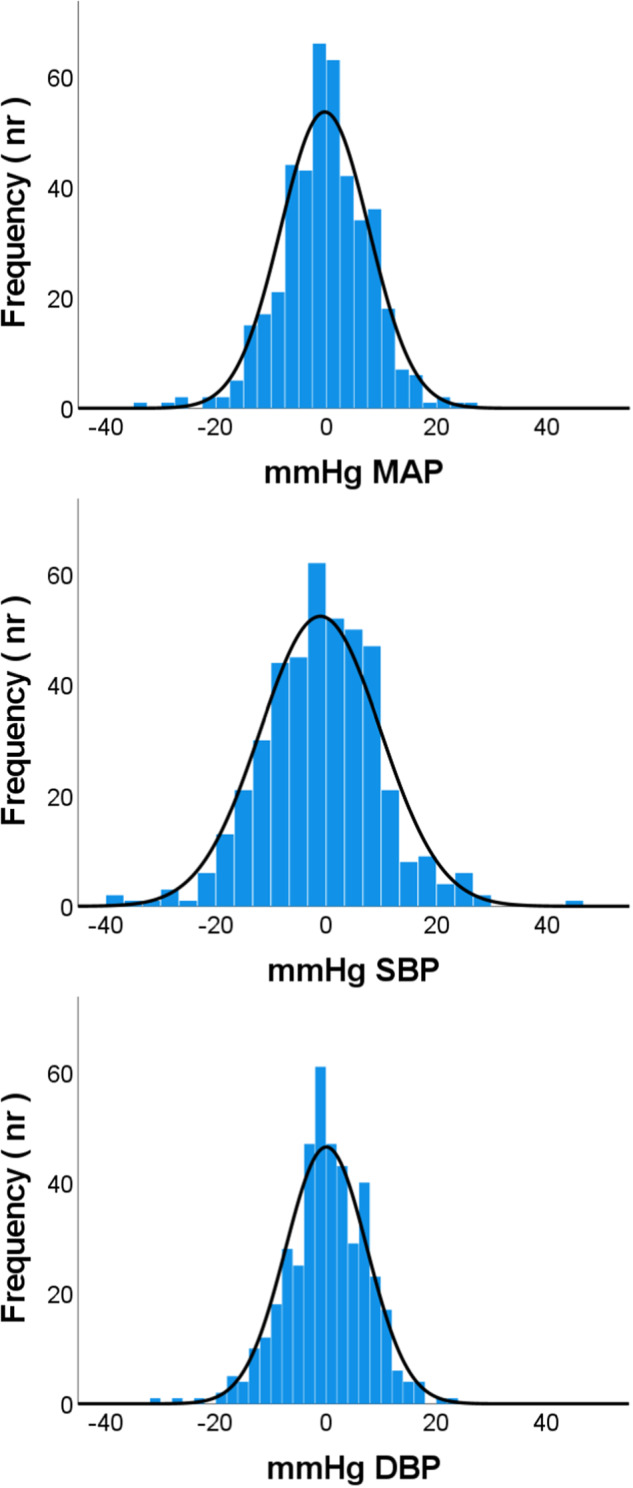
Distribution of difference in 24 h averaged blood pressure between atrial fibrillation and sinus rhythm (AF minus SR) for mean arterial pressure (MAP), systolic blood pressure (BP) and diastolic BP with normality curves displayed. Diff = AF minus SR. Frequency = nr of individuals. SD standard deviation. Diff MAP SD ± 8.0 mmHg, Diff systolic BP SD ± 10.9 mmHg, Diff diastolic BP SD ± 7.4 mmHg

Measures of systolic and diastolic BP, MAP, and PP according to rhythm, are shown in Table 1 and in Fig. 2. Dispersion of data for the intraindividual difference between measurement in AF and in SR during the 24 h is shown in Fig. 3. Systolic BP tended to differ depending on rhythm, with a slightly lower systolic BP with assumed AF. There was a small difference in diastolic BP during night-time measurements, with higher diastolic BP during assumed AF. MAP was not associated to rhythm during any time-period of the 24-h. PP was associated to rhythm, with more prominent differences during night. These differences were, however, small in absolute values, and with generally small effect sizes.

**Table 1:** Blood pressure and heart rate values in AF and SR during 24 h, daytime and night-time ABPM measurements

*n*	24 h AF	24 h SR	*p*	day AF	day SR	*p*	night AF	night SR	*p*
	430			410			291		
SBP	123.6 ± 13.9	124.7 ± 16.1	0.05	125.9 ± 14.1	126.7 ± 14.9	0.10	119.0 ± 18.1	120.0 ± 18.8	0.14
DBP	69.0 ± 8.8	68.9 ± 9.7	0.80	71.0 ± 9.1	71.1 ± 9.4	0.80	64.6 ± 10.9	63.3 ± 10.4	0.01
MAP	87.2 ± 9.5	87.5 ± 10.6	0.47	89.3 ± 9.7	89.6 ± 9.9	0.36	82.8 ± 12.3	82.2 ± 12.1	0.26
PP	54.6 ± 10.9	55.7 ± 12.8	0.002	55.0 ± 11.3	55.6 ± 12.3	0.06	54.4 ± 13.0	56.7 ± 14.0	<0.001
ARV SBP	10.0 ± 3.2	9.9 ± 3.9	0.69	10.0 ± 3.8	10.1 ± 4.2	0.84	10.0 ± 5.0	9.4 ± 5.6	0.25
ARV DBP	6.8 ± 2.4	6.1 ± 2.6	<0.001	6.8 ± 3.0	6.2 ± 3.1	0.01	6.7 ± 3.0	6.3 ± 3.5	0.19
ARV MAP	6.3 ± 2.0	5.9 ± 2.3	0.02	6.2 ± 2.5	6.0 ± 2.7	0.19	6.4 ± 2.8	6.2 ± 3.0	0.48
CV SBP	8.1 ± 2.4	8.0 ± 3.0	0.53	7.9 ± 2.9	7.9 ± 3.3	0.91	8.4 ± 3.9	7.9 ± 4.8	0.25
CV DBP	9.9 ± 3.3	9.0 ± 3.7	<0.001	9.7 ± 4.0	8.9 ± 4.6	0.01	10.6 ± 4.7	10.2 ± 5.4	0.36
CV MAP	7.2 ± 2.1	6.9 ± 2.6	0.03	6.9 ± 2.7	6.7 ± 3.0	0.27	7.9 ± 3.6	7.7 ± 3.8	0.54
HR	67.4 ± 11.3	67.5 ± 12.1	0.87	69.1 ± 11.8	68.7 ± 11.8	0.35	63.5 ± 11.4	63.0 ± 12.1	0.35

Mean values ±SD

*SBP* systolic blood pressure, *DBP* diastolic blood pressure, *MAP* mean arterial pressure, *PP* pulse pressure, *ARV* averaged real variability, *CV* coefficient of variation, *HR* heart rate

### The influence of heart rate on blood pressure values, according to rhythm

Analyses on BP values were also performed with data separated for measurements with HR <90 and ≥90 bpm. HR ranged from 36–110 during 24 h, 41–110 during daytime and 38–110 during night-time (Table 1). There were lower numbers of individuals with BP episodes during both assumed AF and SR with HR ≥ 90 bpm compared to HR <90 bpm (Table 2), and paired statistics were only performed on groups >100 individuals, the minimum for calculated statistical power. BP values in assumed AF and in SR (Table 2) were numerically similar for measurements with low and high HR.

**Table 2:** Blood pressure values during 24 h, daytime and night-time ABPM measurements according to heart rate <90 or ≥90 bpm

*n*	24 h AF	24 h SR	*p*	day AF	day SR	*p*	night AF	night SR	*p*
	426			407			285		
SBP, HR < 90	123.3 ± 14.0	124.6 ± 16.0	0.02	125.7 ± 14.1	126.7 ± 15.0	0.05	119.0 ± 18.3	120.0 ± 18.5	0.16
DBP, HR < 90	68.7 ± 8.9	68.7 ± 10.0	0.99	70.6 ± 9.1	70.9 ± 9.6	0.38	64.6 ± 11.0	63.2 ± 10.5	0.004
*n*	114		*p*	97		*p*	26		*p*
SBP, HR ≥ 90	125.8 ± 17.8	125.6 ± 19.2	0.86	125.7 ± 17.3	127.6 ± 18.4	n/a	120.1 ± 15.9	119.4 ± 21.7	n/a
DBP, HR ≥ 90	74.5 ± 10.6	74.1 ± 11.5	0.66	74.7 ± 10.6	75.5 ± 10.9	n/a	70.7 ± 10.3	68.1 ± 11.8	n/a

Mean values ± SD for blood pressure according to heart rate measurements <90 and ≥90 bpm

Individuals without BP measurements with HR <90 or ≥90 bpm during the ABPM, or without matched BP measurements with HR <90 or ≥90 bpm in AF and SR were not evaluated

*SBP* systolic blood pressure, *HR* heart rate, *DBP* diastolic blood pressure

### The influence of high vs low BP on rhythm dependent differences in blood pressure

With data separated for 24 h systolic BP < or ≥124 mmHg (average value), the trend for lower systolic BP in AF compared to SR was only seen in measurements with systolic BP higher than average (≥124 mmHg difference −1.3 mmHg, *p* = <0.001 vs <124 mmHg, +0.6 mmHg, *p* = 0.07). For night-time diastolic BP measurements results were also dependent on high vs low BP with larger differences seen with diastolic BP higher than average (≥64 mmHg difference +1.3 mmHg, *p* = 0.006, vs <64 mmHg, +0.6 mmHg, *p* = 0.07).

### Blood pressure variability, according to rhythm

Measures of BP variability, assessed by ARV and by CV of ARV, are shown in Table 1. The variability was higher for diastolic BP and MAP measurements during assumed AF compared to SR, both for ARV and for CV of ARV during 24-h. For daytime measurements, the variability of diastolic BP, but not of MAP, was higher in assumed AF, while diastolic BP variability was unaffected by rhythm during night-time. In contrast, systolic BP variability was not affected by rhythm during any time period of the recording (Table 1). In addition, a linear regression was performed to assess the impact on variability indices by the different number of BP measurements for each individual. However, adding number of measurements or percentage of AF measurements in the analysis did not change the results (data not shown).

### Interaction analyses

There were no interactions for BP according to rhythm with age or sex, while ongoing antihypertensive medication showed an interaction with night-time diastolic BP (*p* = 0.03). This was due to a greater fall in diastolic BP from assumed AF to SR during night-time in those without antihypertensive treatment.

## Discussion

This real-life study in an out-of-hospital setting investigated the performance of an ABPM device with an algorithm for AF detection (Microlife WatchBP 03 AFIB) in 430 mostly hypertensive subjects. For the first time intraindividual comparisons of BP measurements in adjacent episodes of assumed paroxysmal AF and in normal rhythm were assessed during a full ABPM registration. We found that averaged MAP was similar, averaged systolic BP tended to be lower, and diastolic BP higher in assumed AF, however with absolute differences of limited clinical relevance.

As compared to sinus rhythm, we observed slightly lower systolic BP and slightly higher diastolic BP values in assumed AF, rendering PP values to be dependent on rhythm in this study using intraindividual comparisons. This is in line with our previous findings comparing patients with high likelihood of AF to individuals with low likelihood of AF [22] and other studies with intraindividual comparisons [13, 14]. However, those studies [13, 14] did not compare averaged BP values in adjacent episodes of AF and normal rhythm during an ABPM as we did in the current study. A lower systolic BP and higher diastolic BP seems to be a systematic error of cuff-measured BP, since similar findings are apparent also in SR, using invasive measurements as reference [33, 34]. Compared to invasive measurements, these errors seem to be larger in AF than in SR [17]. Our results imply that using averaged values of MAP, which is the actual measure with oscillometric devices [35], provides more accurate information than the derived values of systolic and diastolic BP when using the oscillometric technique during AF. This notwithstanding, the differences for systolic and diastolic BP values between AF and SR were small (<1.5 mm Hg), suggesting little impact on clinical decision making.

Our findings do not appear to be critically dependent on HR during daily life (using a partition value of 90 bpm). Few studies have investigated the reliability of BP measurements in different HR strata. Findings by Xie et al. suggested larger differences between invasive and non-invasive oscillometric BP measurements with higher HR in AF [17]. In that study, the correlations between differences in invasive and oscillometric BP and HR were linear [17]. Our data were obtained in an out-of-hospital setting where the observed maximum HR was 110 bpm, whereas Xie et al. investigated a large part of the AF patients during tachycardia, with a maximum HR of 150 bpm [17]. Also Zhao et al. [32] showed larger differences in BP with HR values beyond 120 bpm. This might explain the inconsistent findings. Thus, we suggest that BP measurement by the oscillometric technique can be reliable also in AF with a HR up to approximately 110 bpm.

However, a factor that seemed to induce larger BP differences between AF and SR in our study was a BP higher than the average for the population. Even if differences were small in absolute numbers, this indicates that high BP might be a factor for rhythm dependent BP differences during daily life.

In the present study, diastolic (but not systolic) BP variability was dependent on rhythm, with a higher variability for diastolic BP in assumed AF. ABPM variability has important prognostic implications [31, 36]. The mechanisms for this association are not fully understood but sympathetic activation and impaired baroreflex sensitivity seem to be of importance [30, 37]. It has also been reported that systolic, but not diastolic, BP variability correlates to carotid-femoral pulse wave velocity (i.e., aortic stiffness) [37]. In line with this, systolic BP variability seems to be more closely associated with future cardiovascular risk than diastolic BP variability [31]. An increased BP variability per se is a risk factor for new onset AF, and visit-to-visit BP variability seems to be of importance for outcome in AF patients [38]. Patients in AF also have higher invasively measured beat-to-beat variability than subjects without arrhythmia for both systolic and diastolic BP, with a larger difference for diastolic than for systolic BP variability [39]. Taken together, our results of rhythm dependent diastolic but not systolic BP variability by intraindividual comparisons may be taken to suggest that diastolic BP variability is affected more by rhythm, whereas systolic BP variability, reflecting arterial stiffness, might be increased in an AF population independent of the rhythm during BP measurements.

There are some strengths with this study. To our knowledge, this is the first study with real-life data in an out-of-hospital setting on the performance of ABPM monitoring in AF. We are also first to use episodes with assumed paroxysmal AF and adjacent episodes in normal rhythm for intraindividual comparisons of averaged BP during a complete ABPM registration. Interaction analyses of sex, age and antihypertensive medication suggest that our findings are valid for a broad population of hypertensive patients. Our results point towards the feasibility of using ABPM for clinical decision making also in patients with AF.

There are also important limitations to this study to consider. First, for the validation of true AF we cannot rule out that measurements during very frequent and long episodes of supraventricular ectopic beats (SVEBs) were included in error, since we did not have simultaneous ECG recordings. However, previous validation studies of this device (with even less strict criteria applied than ours) showed an excellent pooled specificity of 94% [8]. The remaining proportion of false positives were almost exclusively SVEBs [8], where excessive supraventricular ectopic activity is closely related to both AF and to future cardiovascular events [40]. Excessive supraventricular ectopic activity is usually defined as 30 premature atrial contractions per h (i.e 0,8% per h with a HR of 60 bpm) or a run of >20 premature atrial contractions (corresponding to less than 30 s) [40]. Considering our definition of assumed AF to require >30% of the time in assumed AF and episodes with a duration >30 min, makes the potential confounding of our results from premature atrial contractions limited. Second, some individuals displayed larger differences in BP between AF and SR (Fig. 3), however dispersed with a normal distribution. Larger differences were not clearly related to high vs low HR in our study, but seemed to be associated with a BP above the average for the study population. Further studies are needed to better understand the characteristics of these individuals. Third, the selection of patients among elderly mainly hypertensive subjects from community pharmacies may limit the generalizability of our findings.

In conclusion, this is an out-of-hospital study in mostly hypertensive subjects on the performance of an ABPM device with an algorithm for AF detection. We assessed for the first time intraindividual comparisons of averaged BP measurements in adjacent episodes of assumed paroxysmal AF and normal rhythm during an ABPM registration. Compared to SR, averaged MAP was similar, averaged systolic BP tended to be lower, and diastolic BP higher in assumed AF. Results were not critically dependent on HR < or ≥90 bpm but BP higher than average might have induced larger rhythm dependent differences. However, the absolute BP differences were small and suggests little impact on clinical decision making. Our results imply that ABPM is feasible and informative also in patients with AF. In addition, an AF detection algorithm offers a new approach to evaluate the reliability of averaged BP values during AF by intraindividual comparisons to normal rhythm during an ABPM registration.
